# Adaptive Esports for People With Spinal Cord Injury: New Frontiers for Inclusion in Mainstream Sports Performance

**DOI:** 10.3389/fpsyg.2021.612350

**Published:** 2021-04-15

**Authors:** Laura Tabacof, Sophie Dewil, Joseph E. Herrera, Mar Cortes, David Putrino

**Affiliations:** Department of Rehabilitation and Human Performance, Icahn School of Medicine at Mount Sinai, New York, NY, United States

**Keywords:** spinal cord injuries, video games, sports for persons with disabilities, community participation, social isolation

## Abstract

**Introduction:** People with Spinal Cord Injury (SCI) are at risk of feeling socially disconnected. Competitive esports present an opportunity for people with SCI to remotely engage in a community. The aim of this study is to discuss barriers to esports participation for people with SCI, present adaptive solutions to these problems, and analyze self-reported changes in social connection.

**Materials and Methods:** We presented a descriptive data collected in the process of a quality improvement initiative at Mount Sinai Hospital. In 2019, seven individuals with cervical SCI and quadriplegia participated in a special interest group on esports. Group scores were then analyzed for evidence of between subjects variability using a single sample *t*-test. A Pearson's correlation was conducted to determine the relationship between social connectedness and demographic data.

**Results:** All players experienced functional limitations as a result of their injury but managed to design personalized gaming setups with adaptive equipment that allowed them to successfully compete in esports. All players reported a positive change in perceived social connectedness (*p* < 0.001) after participating in the special interest group. Score on Social Connectedness Scale negatively correlated with Time since injury (years).

**Discussion:** It is feasible to create adaptive gaming setups that can be used by people with differing degrees and severity of SCI in a competitive esports environment. Technology and adaptive competitive esports have a potential to improve social connectedness and inclusion in people with quadriplegia. Further research on efficacy and effectiveness of these inclusive environments and their effects on quality of life, activity, and participation is warranted.

## Introduction

The National Spinal Cord Injury Statistical Center estimates that there are a total of 291,000 people living with Spinal Cord Injury (SCI) in the United States of America and another 17,730 spinal cord injuries occur every year (National Spinal Cord Injury Statistical Center, 2019). Life after SCI often involves a significant change in lifestyle due to the changes in physical capabilities that are brought about by the injury. In addition, secondary medical conditions that occur due to SCI significantly affect all aspects of life, including employment, mobility, independence and dignity (Post and Noreau, 2005). This issue is compounded by the fact that access to appropriate medical and social support services is often limited for people with SCI (Stillman et al., 2014).

Social support and employment are consistently rated by people with SCI as important for successful post-injury functioning (Bamford et al., 1986; Krause, 1992; Kennedy et al., 2006). Feelings of loneliness and social isolation weigh heavily on many individuals with SCI, and can often contribute to negative health and well-being outcomes (Barclay et al., 2016; Tsai et al., 2017). Work by Guilcher et al. (2019) has shown that the size of one's social network is uncorrelated to feelings of loneliness, rather the frequency and quality of authentic social interactions were the most important factors for reducing loneliness and social disconnectedness. As a result of the global SAR-CoV-2 pandemic, mandated social distancing has intensified the social isolation of high health-risk individuals, and healthcare and social support accessibility is of greater concern than ever before (Jumreornvong et al., 2020).

Competitive organized online gaming (i.e., *esports*) has been steadily growing in popularity since the first esports competition occurred in 1972. In the decades following, esports leagues, competitions, and tournaments have been increasing in frequency, skill, and competitiveness. In recent years, esports has transcended the status of “hobby” for many of its participants to be now considered a serious activity with a professional participatory element and resulting in the formation of multiple distinct subcultures (Seo and Jung, 2016). Online gaming involves trust, respect, and cooperation, and these foundational elements have fostered a global and inclusive community of gamers (Freeman and Wohn, 2017).

Universal Design (UD) is a guiding principle developed to promote equal access to products, environments, and services (Lid, 2014). Many individuals with physical differences wish to engage in esports but are unable to do so due to lack of accessible technology. Previous work describing video-game interventions in individuals with spinal cord injury focuses on gamified rehabilitation interventions eliciting cardiorespiratory fitness response (O'Connor et al., 2000; Burns et al., 2012) or enhancing muscle activation (Sayenko et al., 2011; Jaramillo et al., 2019). However, the exploration of esports in a non-therapeutic, recreational context, and its role in promoting social connectedness and reducing isolation has not been reported and is a novel aspect of this quality improvement (QI) initiative.

The purpose of this study is to identify specific issues that have hindered participation in esports activities in seven individuals with quadriplegia secondary to SCI. Additionally, we examine the different ways that these individuals have created (or plan to create) adaptive environments to overcome these issues and enable more active participation in the esports community. Finally, we evaluate whether the recent inclusion of these players in esports activities has changed their perception of their own social isolation.

## Materials and Methods

### Settings and Participants

We present results from a quality improvement initiative to enhance social support services for the SCI patient population at Mount Sinai Hospital.

Within the hospital system there is a social support infrastructure in place to assist patients with spinal cord injury (SCI) in reintegrating into society after their injury. Within the main support groups, consisting of up to 30 active members, a number of participants expressed interest in esports and video gaming. These participants, in partnership with the study team, formed a special interest group on esports and people with SCI. Individuals were eligible to participate in this group if they had cervical spinal cord injuries (SCI), were participating in a social support group for people with SCI at Mount Sinai and had sufficient degree of cervical motor control (rotation and/or flexion/extension) and range of motion to use the adaptive gaming devices. There were no specific exclusion criteria.

As part of the special interest group, participants worked with the study team to create personalized gaming setups and participated in online and offline gaming sessions. These sessions were conducted individually or as part of group practice. All seven members of the special interest group were contacted to engage in this QI initiative, and all seven individuals responded. All participants gave verbal permission for the use of the de-identified data that was collected as part of this QI initiative.

### Gaming Customization

Each participant in the focus group had access to a Maingear Vybe Desktop PC, MSI Optix MAG27C, Logitech G703 Lightspeed wireless gaming mouse, and Logitech G915 Lightspeed wireless gaming keyboard. Additionally, each player was given access to adaptive equipment that could meet their needs, including the Xbox adaptive controller (Microsoft Corporation, Redmond, WA) and an accompanying Logitech G Adaptive Gaming Kit (Logitech International S.A., Lausanne, Switzerland), the “QuadStick” controller (QuadStick, Great Falls, MT), Tobii Eye X eye tracking technology (Tobii AB, Stockholm, Sweden) and Warfighter Engaged joysticks (Warfighter Engaged, Inc., Wayne, NJ). Each player then created a custom gaming setup for optimal participation in esports competition. Gaming environment setups were continuously optimized based on qualitative feedback from players and quantifiable success at selected games. Need for specific optimizations was generally dictated by each player's functional capacity and level of injury, and thus was highly individualized and required specific customizations. The need for equipment upgrades to enhance gaming ability was continuously assessed and addressed. All players had access to a clinical coordinator from the hospital to assist with equipment setup and/or technical support on request.

### Data Collection and Outcome Measures

Study data were collected over phone and video calls with members of the study team, and via SurveyMonkey (SurveyMonkey Inc., San Mateo, CA). Baseline demographics included age, gender, SCI level (ASIA's score) (Kirshblum et al., 2011), time since SCI, level of independence (SCIM III), and past gaming experience. Outcome measures included Social Connectedness Scores (SCS), perception of change in social connectedness, current gaming setup, opinions on current gaming setup, and ideal gaming setup (Table 1).

**Table 1:** Phone interview questionnaire.

1.When did you start gaming?
2.What gaming setup have you traditionally used?
a.Why do you like it?
b.Why do you dislike it?
3.Are you using the same setup now?
a.Why do you like it?
b.Why do you dislike it?
4.Have you used the keyboard from Logitech?
a.Why do you like it?
b.Why do you dislike it?
5.Have you used the mouse from Logitech?
a.Why do you like it?
b.Why do you dislike it?
6.What is your ideal setup
a.Why do you like it?
b.Why do you dislike it?

To evaluate participant's level of function and independence, we used The Spinal Cord Independence Measure (SCIM III). The scale evaluates three domains of function: self-care (score range 0–20), respiration and sphincter management (0–40), and mobility (0–40). The total score ranges from 0 to 100 where higher scores indicate higher levels of independence (Itzkovich et al., 2018). SCI level was classified according to the American Spinal Injury Association (ASIA)'s International Standards for Neurological Classification of Spinal Cord Injury (ISNCSCI) (Kirshblum et al., 2011). The scale describes the neurological level of injury and five impairment classification levels (A, B, C, D, and E) ranging from complete loss of neural function in the affected area (A) to completely normal (E) (Table 2).

**Table 2:** Player demographics, clinical details, and social connectedness data.

	**Age**	**Gender**	**NLI**	**AIS**	**Time since injury (years)**	**SCIM: self-care**	**SCIM: respiration and sphincter management**	**SCIM: mobility**	**SCS**	**Perception of change in social connectedness**
Player 1	29	F	C3	A	11	0	13	0	41	1
Player 2	36	M	C7	A	5	10	17	10	48	1
Player 3	43	M	C4	D	18	10	29	11	46	2
Player 4	30	M	C5	B	13	14	35	14	48	1
Player 5	39	M	C5	A	25	6	19	4	25	2
Player 6	45	M	C3	A	15	0	13	0	40	2
Player 7	34	M	C5	A	12	9	29	2	45	2

*ASIA, American Spinal Injury Association (ASIA)'s International Standards for Neurological Classification of Spinal Cord Injury (ISNCSCI); NLI, ASIA Neurological Level of Injury; AIS, ASIA Impairment Score; SCS, Social Connectedness Scale (SCS); SCIM, Spinal Cord Independence Measure*.

*The AIS has five classification levels, ranging from complete loss of neural function in the affected area to completely normal: (A) the impairment is complete and there is no motor or sensory function left below the level of injury. (B) The impairment is incomplete and sensory function (but not motor function) is preserved below the neurologic level while some sensation is preserved in the sacral segments. (C) The impairment is incomplete and motor function is preserved below the neurologic level, but more than half of the key muscles below the neurologic level have a muscle grade <3/5. (D) The impairment is incomplete and motor function is preserved below the neurologic level, and at least half of the key muscles below the neurologic level have a muscle grade of 3/5 or more. (E) The patient's functions are normal and all motor and sensory functions are preserved*.

Perception of connection to others in their social environment was self-reported using the Social Connectedness Scale (SCS). The scale presents 8 negative perception statements (i.e., “I feel disconnected from the world around me”) rated on a 6 point Likert scale ranging from 1 (strongly agree) to 6 (strongly disagree). Scores were summed with a potential range of 8–48 with a higher score denoting a higher level of perceived social connectedness (Lee and Robbins, 1995) (Table 2).

To evaluate players' perception of change in social isolation and social connection, players were presented with the statement “Since becoming involved in an adaptive esports team, I feel less socially isolated and more socially connected” and asked to rate their agreement on a 5-point Likert scale ranging from 1(strongly agree) to 5 (strongly disagree) (Table 2).

### Data Analysis

Statistical analysis was conducted on the “perception of change in social isolation” data using MATLAB (MathWorks, Inc.). Likert scale data were analyzed by normalizing responses around a zero score for the neutral “not sure” response, a two point score for a “strongly agree” response and a minus two point score for a “strongly disagree” response. Group scores were then analyzed for evidence of a significant non-zero mean using a single sample *t*-test; effect size was calculated using Cohen's d. Associations between demographic, clinical, and social connectedness information were conducted in R (R Core Team, 2017) using a Pearson's correlation.

## Results

### The Special Interest Group

Between January and December of 2019, seven individuals (age: 29–45; male/female: 6/1) with quadriplegia participated in a special interest group on esports. Details on neurological level of injury, ASIA impairment score and time since injury can be found in Table 2.

All participants presented motor impairments in both upper and lower extremities. Two individuals had high level complete injuries and were fully dependent for feeding, bathing, dressing, grooming, toilet use, transfer, and mobility. All individuals were able to breathe independently. Six of the seven individuals had motorized wheelchairs while one was fully dependent for mobility in a manual wheelchair.

### Gaming Setups

Most participants (*n* = 5) had previous experience playing video games but none had played competitively. All players managed to create a gaming setup that allowed them to successfully play a wide variety of games. Players engaged in a variety of different games including Fortnite, Rocket League, and Call of Duty. Players had the option to play games independently or online with the remainder of the team.

#### Player One (P1)

##### History

Player One is a 29-year-old woman with an 11 year history of C3 ASIA A SCI. P1 has limited cervical movements and no upper limb or lower limb movements due to her high level of injury.

##### Prior Gaming Experience

Prior to joining the special interest group, P1 did not engage in any esports activities as she was unaware of options that were suitable for her adaptive needs.

##### Adaptive Gaming Experience

**Figure 1:**
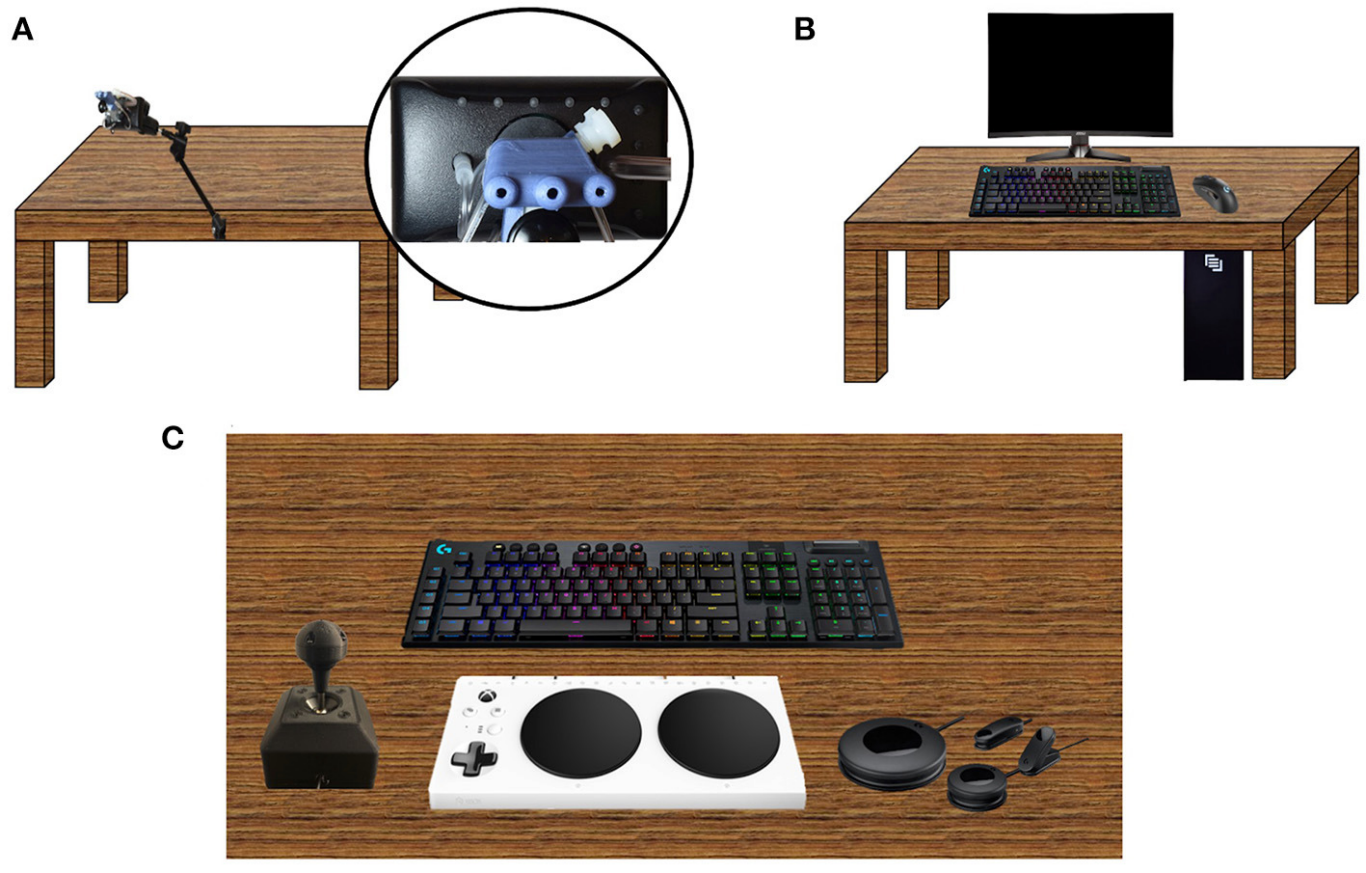
Gaming setups. **(A)** Gaming setup of P1 and P6 using a QuadStick device. The Quadstick requires only the use of the head and neck. The QuadStick utilizes a coded mapping of sips and puffs to controller commands on a gaming system (Xbox, Playstation, or PC). There are three main tubes into which a user can sip or puff. Each sip or puff corresponds with a command on the gaming system and is controlled through oral orbicularis and diaphragmatic muscle activation. The sip and puff tubes are mounted on a joystick that is controlled through cervical movement (rotation and flexion/extension). The QuadStick sensor sensitivity can be tailored according to each individual's neck movement and sip/puff intensity. The joystick movement corresponds with movement of the player's character in the game. Multiple different controller mappings can be uploaded to the QuadStick at one time and the user is able to dynamically switch between them through the use of a side sip and puff tube. See Supplementary Figure 1 for an example of the button mapping used by the two QuadStick players presented here. **(B)** Original gaming setup of P2, P3, P5, and P7 using the Logitech keyboard and mouse with the MSI Optix screen **(C)** P4's current gaming setup. This setup includes a combination of the Xbox Adaptive controller, the Logitech Adaptive Gaming Kit, one joystick from Warfighter Engaged, and the Logitech keyboard.

P1's preferred esports environmental setup is a QuadStick connected to the Maingear Desktop PC (Figure 1A). She plays the game while sitting in her wheelchair with the need of neck support. She uses her cervical control and range of motion (rotation and flexion/extension) to move the joystick. The joystick movement corresponds with movement of the player's character in the game. Player 1 uses her oral orbicular and diaphragmatic muscle control to sip and puff on the three mounted tubes of the QuadStick.

#### Player Two (P2)

##### History

P2 is a 36-year-old male with a 5 year history of C7 ASIA A SCI.

##### Prior Gaming Experience

After his injury, P2 was already engaged in esports activities using PlayStation console and a standard PS4 controller, but reported significant functional difficulties with hand grip and release, as well as participation in games that required individual finger movements. This limited his enjoyment of esports-related activities.

##### Adaptive Gaming Experience

Since joining the esports special interest group, P2's initial esports environmental setup was a Maingear desktop gaming PC using the Logitech keyboard and mouse (Figure 1B). However, P2 experienced initial difficulties with this setup, reporting that the “sleek design [of the mouse] makes it very difficult to handle. [It] easily slips from my hand.” He enjoyed using the keyboard but found the keys too small to be practical for him, particularly with the more complex games requiring fast reactions such as Fortnite. He explained, “I think it's just the fact that Fortnite has so many different commands to use that it becomes difficult for somebody with limited use of the fingers to use the keyboard.”

**Figure 2:**
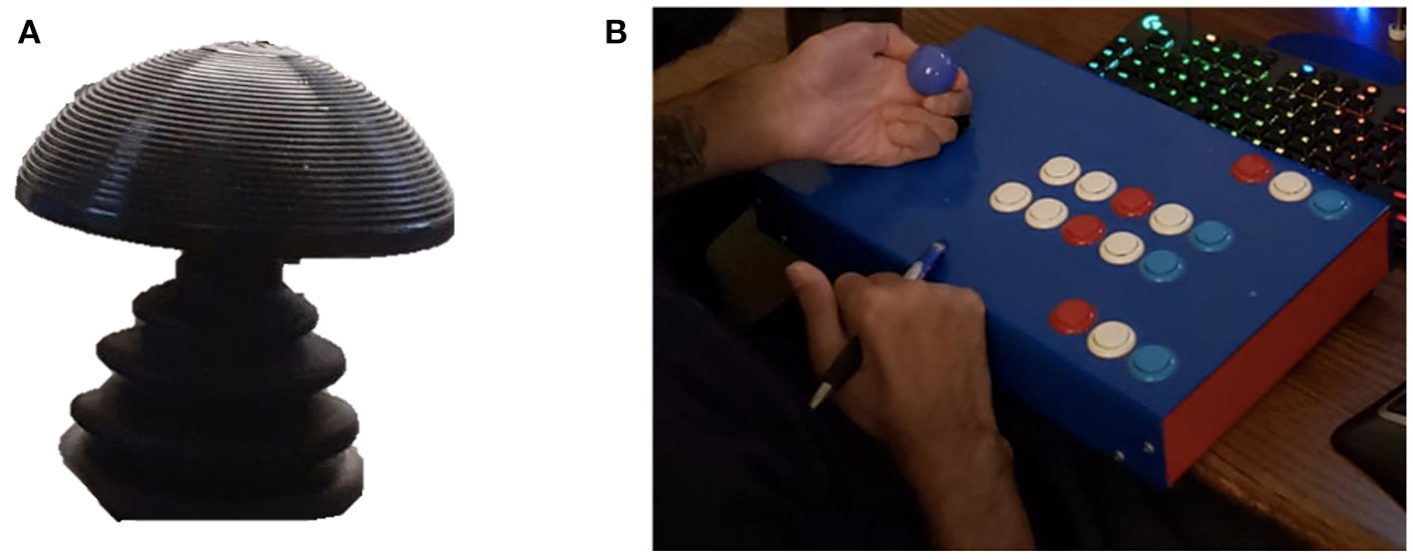
**(A)** The joystick from P2's powered wheelchair, with a height of 2.5 cm and a diameter of 6.5 cm. **(B)** P5's custom controller.

**Figure 3:**
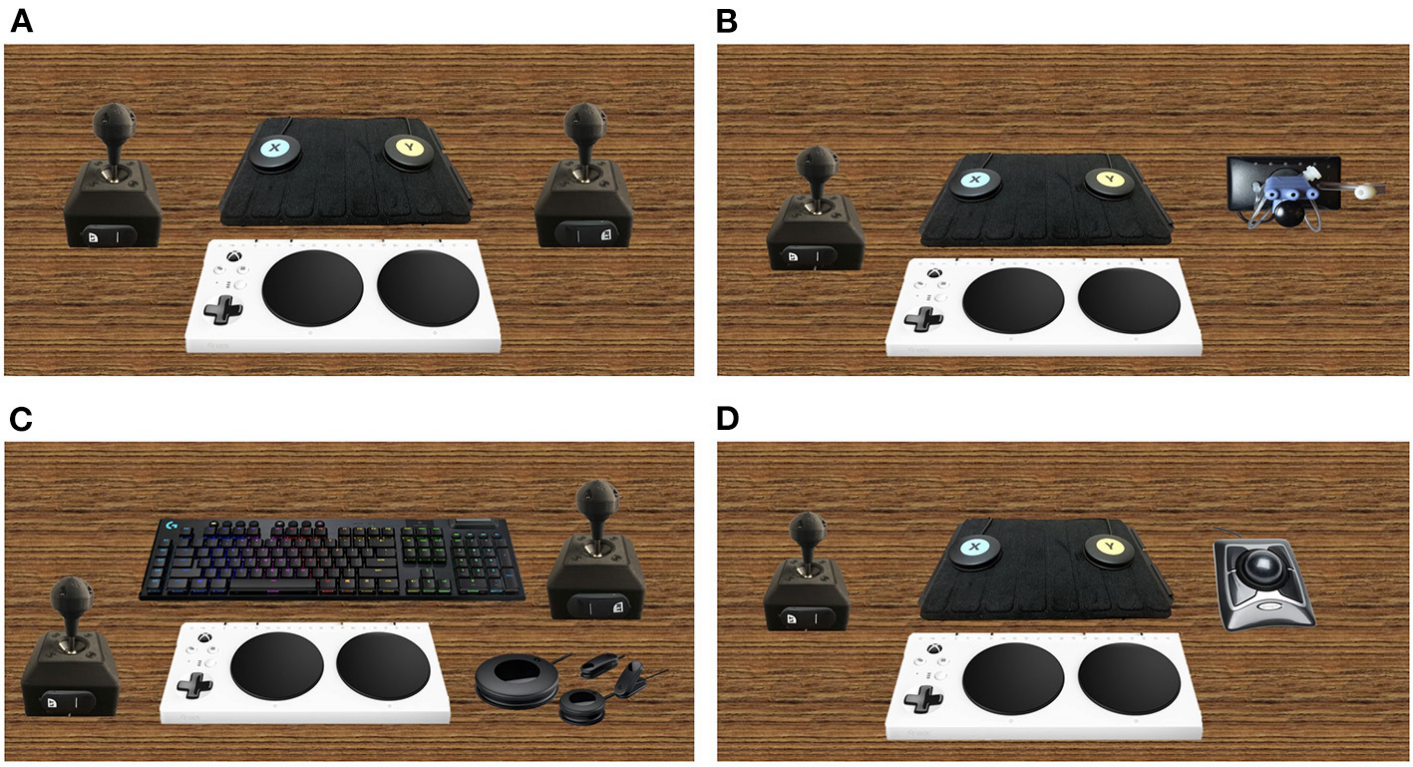
Ideal or planned gaming setups. **(A)** Ideal setup of P2 and P7. This setup includes the adaptive Xbox controller, the Logitech Adaptive Gaming Kit, and two joysticks from Warfighter Engaged with an analog switch from the Adaptive Gaming Kit on the base of each joystick. **(B)** P3's ideal gaming setup. This setup includes the adaptive Xbox controller, the Logitech Adaptive Gaming Kit, one joystick from Warfighter Engaged with an analog switch from the Adaptive Gaming Kit on the base, and one QuadStick. **(C)** P4's current gaming setup. This setup includes a combination of the Xbox Adaptive controller, the Logitech Adaptive Gaming Kit, one joystick from Warfighter Engaged, and the Logitech keyboard **(D)** P5's ideal gaming setup. This setup includes the adaptive Xbox controller, the Logitech Adaptive Gaming Kit, a Warfighter Engaged joystick with an analog switch from the Adaptive Gaming Kit on the base, and a trackball mouse.

P2 worked with staff at the ARC to develop a gaming setup more tailored to his individual needs. Inspiration for his ideal setup came from existing equipment that he currently uses with ease and precision. For instance, P2 uses a powered wheelchair for mobility that is controlled by a ball-shaped joystick (Figure 2A). He has an expert ability to navigate complex environments in his chair using this joystick, and expressed interest in creating a gaming joystick with a design that mirrored his chair's joystick. P2 is currently designing a personalized gaming setup that is based around the main module of the adaptive Xbox controller, as well as the accompanying accessories kit. In addition, he is collaborating with Warfighter Engaged to create two joysticks of the same dimensions as his chair joystick for him to use while gaming. He plans to attach a small analog switch from the Xbox adaptive accessories kit to the base of the two joysticks that he can press with his palm while playing. This will allow him easy access to the most vital controls during gameplay (Figure 3A).

#### Player Three (P3)

##### History

P3 is a 43-year-old male with an 18 year history of C4 ASIA D SCI.

##### Prior Gaming Experience

P2's initial esports environmental setup was a Maingear desktop gaming PC using the Logitech keyboard and mouse (Figure 1B). Before his injury, P3 played video games with a PlayStation console and controller. After his injury he found the PlayStation controller to be too small, making it difficult for him to use tactile feedback to discriminate between different buttons. He soon transitioned to an Xbox controller, finding the larger controller to be more conducive to his needs. P3 enjoyed playing with this configuration for years, but still felt limitations in his overall esports ability due to the inadequacy of the equipment. In particular, P3 identified lower mobility in his right index finger, as a significant barrier for operating a standard gaming controller, and he reported finding it difficult to use the trigger buttons on the controller. As a result, P3 often ended up using only his thumbs during a game. He developed a successful rhythm and routine that allowed him to play that way, but expressed interest in using adaptive technology to enhance his setup.

##### Adaptive Gaming Experience

P3 was familiar with the QuadStick as he used the controller for simple computerized activities, such as lifting his hospital bed and turning on the television immediately following his injury. Through physical and occupational therapy, he was able to regain some motor function, and thus transitioned away from using the QuadStick. With a renewed interest in adaptive gaming, P3 has expressed interest in finding a way to integrate the QuadStick into his gaming setup. He appreciates the flexibility that the “sip-and-puff” control functionality allows him, and believes that using that in conjunction with a joystick and the Xbox adaptive controller would provide him with the ideal setup.

More specifically, P3 finds his playing to be most limited by the movement of his left and right pointer fingers, as well as his right hand more broadly. In traditional first person shooter (FPS) games, the right thumb moves a joystick to control the movement of the player's field of view, the left pointer finger aims a weapon, and the right pointer finger presses a trigger to shoot the weapon. P3 plans to use the QuadStick to aim and shoot a weapon. He plans to combine the QuadStick with a joystick that he can use with his left hand, and larger buttons from the Xbox adaptive controller that he can press with his right hand (Figure 3B).

#### Player Four (P4)

##### History

P4 is a 30 year old male with a 13 year history of SCI C5 ASIA B.

##### Prior Gaming Experience

He has been playing video games since he was a young child, using various gaming systems such as a Sega Genesis, a Nintendo Gameboy, and a PlayStation 4. Before joining the Quad Gods, P4 primarily used his PS4 to game. He explained, “I normally rest my PS4 controller on my chest or on my lap. When I play, I always use L1 to aim and R1 to shoot; that is more ideal for me because of my injury.” In contrast, the traditional PS4 control settings use L2 and R2 to aim and shoot. Because of the position that P4 held his controller in, those buttons were inaccessible to him.

##### Adaptive Gaming Experience

P4's adaptive gaming setup utilizes the Maingear desktop PC, accompanying Logitech keyboard and mouse and the Xbox adaptive controller with the accompanying Adaptive Gaming Kit (Figure 1C). P4 reported high satisfaction with the Logitech keyboard and mouse. He specifically appreciated the programmable G-keys that allowed him to save important key combinations; this has allowed him to perform complicated maneuvers in-game that he would otherwise be unable to execute. In addition to his keyboard and mouse setup, P4 has also created a personalized setup using the Xbox adaptive controller and accessories kit, and one controller from Warfighter Engaged. He reported positively on the ease of mobility that the joystick provides him, but stated the need for two joysticks in order to effectively play most games with this setup. P4 plans to obtain a second joystick from Warfighter Engaged and attach an analog switch to each of them for integration into his adaptive setup (Figure 3C).

#### Player Five (P5)

##### History

P5 is a 39 year old male with a 25 year history of C5 ASIA A SCI.

##### Prior Gaming Experience

P5 began playing video games as a child, but after his injury he was unable to use a traditional controller. P5 designed and oversaw the fabrication of his own custom controller with 16 programmable buttons and one joystick that was designed to support an underhand grip (Figure 2B).

##### Adaptive Gaming Experience

P5 has set up his desktop PC to use the Logitech gaming keyboard and mouse (Figure 1B). P5 prefers using a computer over a gaming console because of the advanced ability to personalize his gaming experience using a PC. However, he acknowledged the bigger financial investment required to purchase an acceptable gaming computer as opposed to a standard gaming console. P5 enjoyed using the Logitech gaming keyboard because of his ability to program the macro buttons. He explained, “this helped me a lot playing Assassin's Creed where I created a macro enabling me to hold a button that allowed me to run.” P5 had extreme difficulty holding the Logitech gaming mouse due to the lack of voluntary movement in his hands and therefore preferred to use a trackball mouse. A trackball mouse requires no grip strength as players can simply rest their hand on the trackball to move it. His ideal setup will combine the Warfighter Engaged solid ball cap joystick in his left hand with a trackball mouse in his right hand. With this setup he could control the direction of a character's body by pushing the joystick to one direction and holding it there with a combination of his body weight and gravity. Additionally, P5 can use the fluidity of the trackball mouse to move his character's gaze smoothly across the playing field. He would also attach an analog switch to the joystick so that he can combine movement with action (Figure 3D). For example, that setup would allow him to aim and shoot a weapon without requiring fine motor movement. He explained, “I have the hand eye coordination, I just don't have the dexterity to do that on a small controller.”

#### Player Six (P6)

##### History

P6 is a 45 year old male with a 15 year history of C3 ASIA A SCI.

##### Prior Gaming Experience

Prior to joining the special interest group, P6 did not engage in any esports activities as he was unaware of options that were suitable for his adaptive needs.

##### Adaptive Gaming Experience

P6 connects a QuadStick to the Maingear Desktop PC in order to play video games. He finds first person shooter (FPS) games to be enjoyable but challenging, as the QuadStick uses only one joystick for both aiming and body movement (Figure 1A).

#### Player Seven (P7)

##### History

P7 is a 34-year-old man with a 12 year history of C5 ASIA A SCI.

##### Prior Gaming Experience

P7 began playing video games when he was 5 years old, alternating between console gaming and computer gaming throughout the years. P7 has a deep love of gaming, but after his injury, he has always had difficulties being able to reach and press all of the buttons on a controller or keyboard.

##### Adaptive Gaming Experience

P7 set up his desktop PC to use with the Logitech gaming keyboard and mouse. He found the keyboard to be user friendly, but stated that the keys protruded a bit too much, and were too easy to press; because of this, he would often unintentionally press keys when reaching for a further key. The mouse was smooth and accurate, but the buttons were difficult for him to press (Figure 1B).

Ideally, P7 aims to create a computer setup that provides him with the same expansive set of controls as a keyboard and mouse does, but with keys and buttons more tailored to his needs. He is currently developing a setup with the Xbox adaptive controller and the Logitech adaptive kit, and plans to add to it two Warfighter Engaged joysticks. He will place an analog switch on the base of each joystick so that he can trigger it with his palm (Figure 3A).

### Social Connectedness

**Figure 4:**
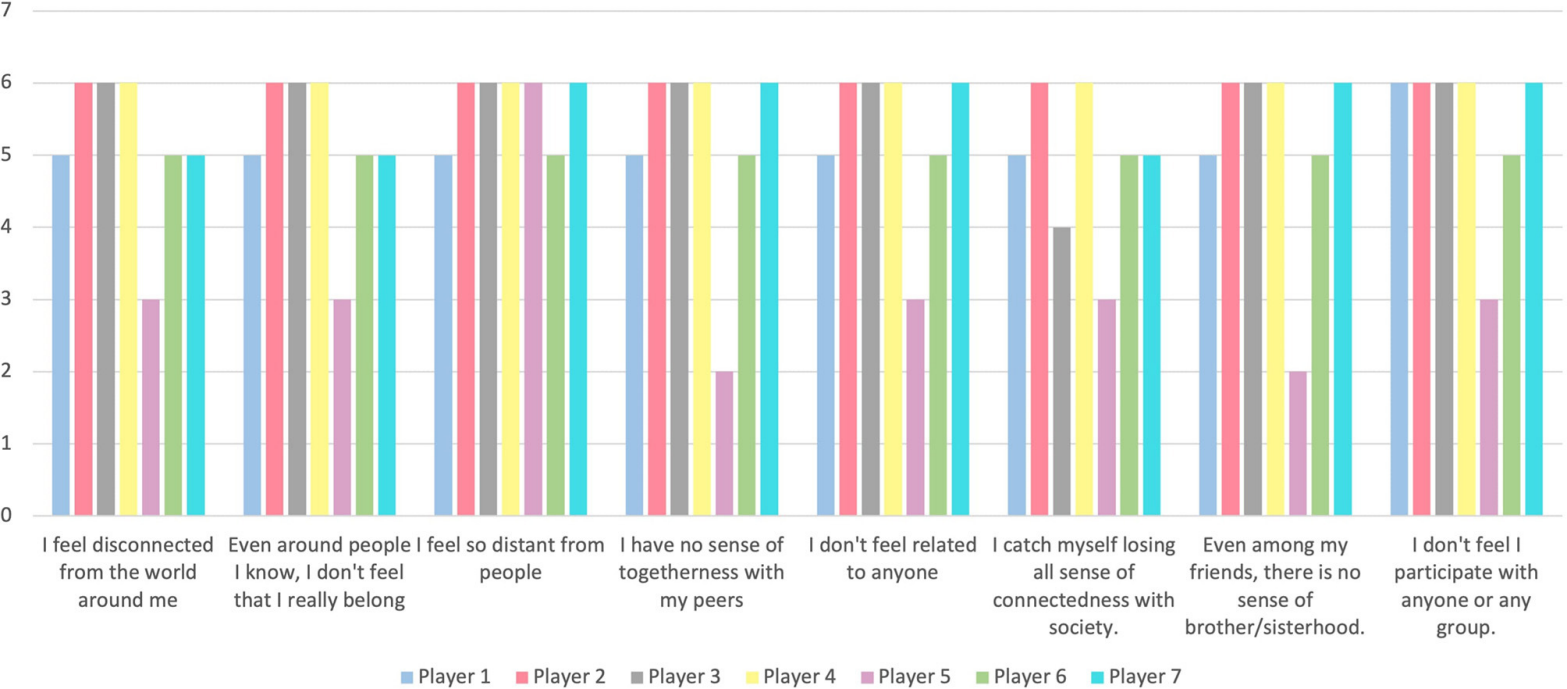
Variability in Social Connectedness Scale (SCS) scores between players by question.

Players responded with an average score of 41.2/48 to the SCS (Figure 4), and 1.57/5 to the question on perceived change in social connection (Table 2). The report on perceived change was significantly non-zero (*M* = 1.57, *SD* = 0.53), *t*_(6)_ = 7.79, *p* < 0.001, and *d* = 2.96.

**Figure 5:**
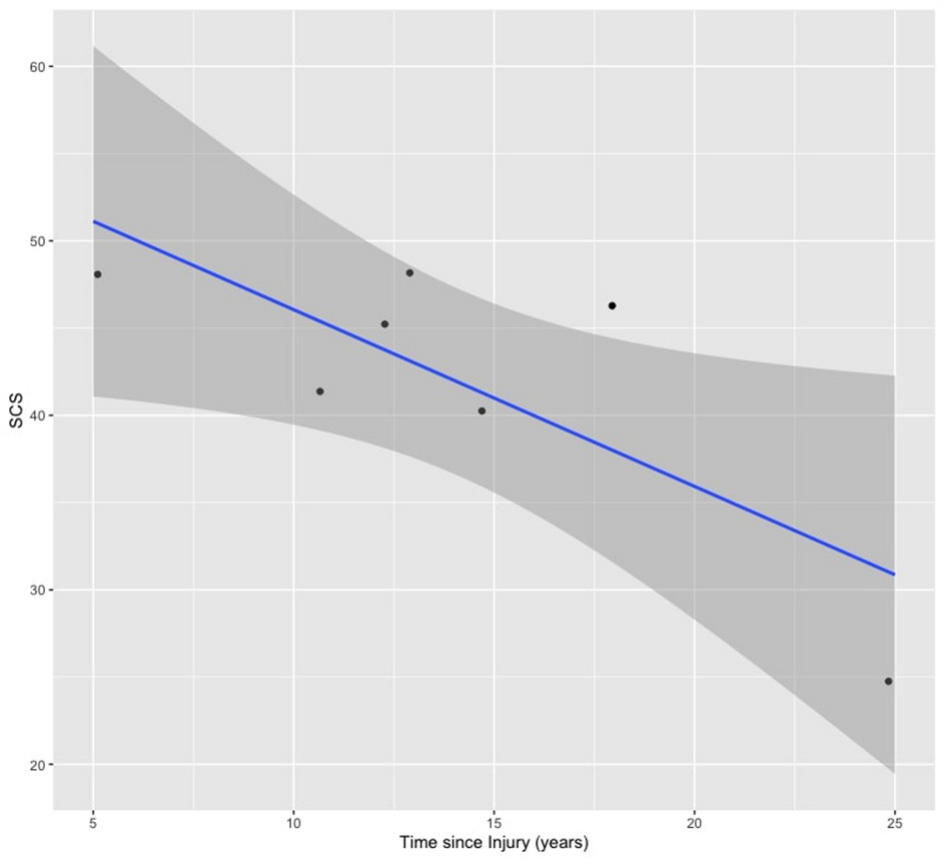
Negative correlation between Time since Injury (years) and SCS scores.

There was a significant negative correlation between Time since injury (years) and the social connectedness score (SCS) [*r*_(5)_ = −0.78, *p* = 0.04]. In other words, longer time since injury was correlated with a lower SCS, and therefore a lower sense of social connectedness (Figure 5). There were no other relationships between SCS or perception of change and other baseline or outcome measures.

## Discussion

To our knowledge, this is the first paper to share adaptive strategies and technologies that enhance, and in some cases permit, full participation in esports for individuals with SCI. In addition, this study identifies common issues with existing esports controller technologies that can prevent full participation in esports for prospective athletes who have physical capabilities that are different to mainstream esports athletes. The aim of Universal Design (UD) is to promote equal opportunity and participation for all people (Lid, 2014), and the findings of this study highlight a critical need to apply UD principles to the development of gaming technology. Notably, the work that has been completed by companies such as Logitech and Microsoft in creating adaptive controller technology demonstrably added value to the experience of the participants in this QI initiative. In addition, the QuadStick was instrumental in permitting participation of two individuals who otherwise could not have engaged in gaming activities because of the severity of their SCI.

Among the surveyed individuals, the most commonly reported motor difficulties were impaired grip strength and lack of individuated finger movements. These motor impairments lead to activity limitations in goal-directed tasks such as pressing buttons, grasping a mouse and smoothly maneuvering a controller, hindering optimal participation in esports. In addition, two individuals with high-level cervical injuries (C3) were completely hindered from playing esports after their injury due to their functional limitations and lack of access to proper adaptive equipment. With the assistance of appropriate technology, all players, regardless of their motor or functional status, were able to competitively participate in esports activities. At the time of writing this manuscript, all of the players continue to actively participate in esports on a daily basis. This work highlights why concepts of UD should be applied to all product design endeavors: accessibility is a fundamental human right (Giannoumis and Stein, 2019), and individuals with motor, cognitive or social/emotional differences should not be limited in their participation in chosen activities. However, the results of this QI initiative also showed that enhanced participation in esports resulted in positive pro-social impact.

All participants in the QI initiative reported that participation in the esports special interest group made them feel more socially connected and less isolated. It is well-established that access to technology is beneficial to people with SCI, as it has the potential to promote independence and foster connection with the general communities (Goodman et al., 2008; Barclay et al., 2016). Moreover, diminished participation in community activities due to SCI physical impairments is associated with a negative impact on mental well-being (Post and Noreau, 2005), emphasizing the need for measures to create maximal inclusion for virtual activities such as esports, especially in the time of COVID-19.

There are notable limitations in the reported data. This study included a small sample with no control group, and as these are QI data from a specific health system, there was no way to ensure recruitment was representative of other cohorts with SCI. Additionally, the lack of baseline SCS scores limited our ability to accurately determine changes in social connectedness. Nonetheless, this work highlighted the capacity of technology to promote inclusion and social connectedness.

We concluded that: (1) It is feasible to create adaptive gaming setups that can be used by people with differing degrees and severity of SCI in a competitive esports environment. (2) Technology and adaptive competitive esports have a potential to improve social connectedness and inclusion in people with quadriplegia. (3) Further research on efficacy and effectiveness of these inclusive environments and their effects on quality of life, activity and participation is warranted.

## Data Availability Statement

The raw data supporting the conclusions of this article will be made available by the authors, without undue reservation, upon request.

## Ethics Statement

Ethical review and approval was not required for the study on human participants in accordance with the local legislation and institutional requirements. Written informed consent for participation was not required for this study in accordance with the national legislation and the institutional requirements.

## Author Contributions

LT and DP conceptualized the work. SD conducted player interviews. SD, LT, and DP performed data analysis and interpretation, and manuscript preparation. All authors read and approved the final version of the manuscript.

## Conflict of Interest

The authors declare that the research was conducted in the absence of any commercial or financial relationships that could be construed as a potential conflict of interest. The handling editor is currently editing co-organizing a Research Topic with one of the authors DP.

## Supplementary Material

Supplementary Figure 1 A sample of the button mapping created for users of the QuadStick to play a shooting game on an Xbox. Column A holds the button representations of the gaming console in use. Column C holds the controls of the QuadStick that are mapped onto the button representations of the gaming console. Column B holds any special formulas for mapping the buttons—none are in place in this example. Column D holds an explanation of the function of each control. This spreadsheet is created in google sheets and loaded into the QuadStick through a USB connection using the QuadStick Manager Program (QMP). The Quadstick is then connected to the gaming console of choice through a USB or bluetooth connection.
