# Supplementary figures and images for: Adaptive Esports for People With Spinal Cord Injury: New Frontiers for Inclusion in Mainstream Sports Performance
Source: Front Psychol. 2021 Apr 15;12:612350. doi: 10.3389/fpsyg.2021.612350 (PMC8082019; doi:10.3389/fpsyg.2021.612350)

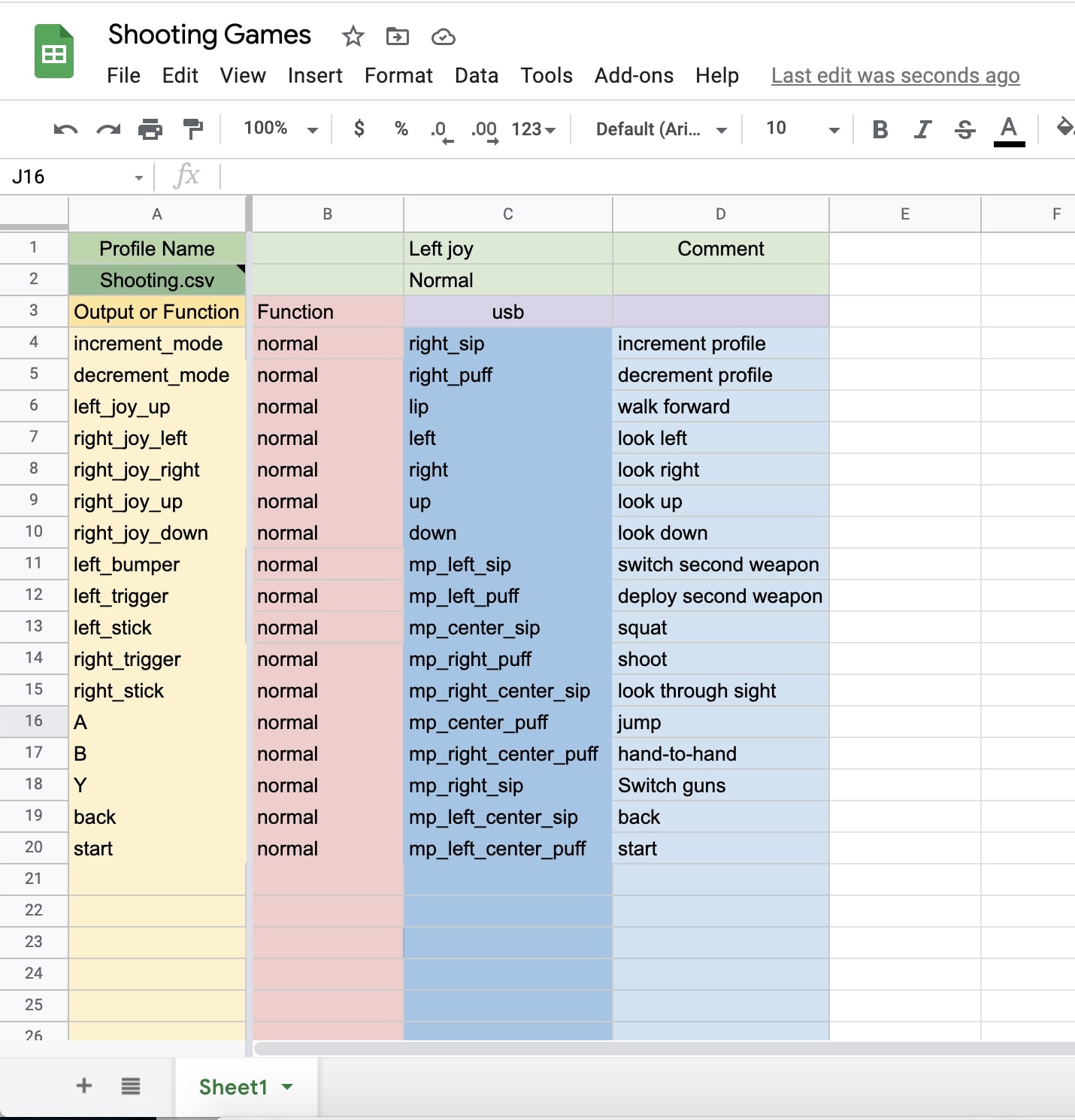

Supplement: Supplementary Figure 1 — A sample of the button mapping created for users of the QuadStick to play a shooting game on an Xbox. Column A holds the button representations of the gaming console in use. Column C holds the controls of the QuadStick that are mapped onto the button representations of the gaming console. Column B holds any special formulas for mapping the buttons—none are in place in this example. Column D holds an explanation of the function of each control. This spreadsheet is created in google sheets and loaded into the QuadStick through a USB connection using the QuadStick Manager Program (QMP). The Quadstick is then connected to the gaming console of choice through a USB or bluetooth connection. [file Image_1.JPEG]
